# SMF-net: semantic-guided multimodal fusion network for precise pancreatic tumor segmentation in medical CT image

**DOI:** 10.3389/fonc.2025.1622426

**Published:** 2025-07-18

**Authors:** Wenyi Zhou, Ziyang Shi, Bin Xie, Fang Li, Jiehao Yin, Yongzhong Zhang, Linan Hu, Lin Li, Yongming Yan, Xiajun Wei, Zhen Hu, Zhengmao Luo, Wanxiang Peng, Xiaochun Xie, Xiaoli Long

**Affiliations:** ^1^ School of Electronic Information and Physics, Central South University of Forestry and Technology, Changsha, China; ^2^ Department of Radiology, Zhuzhou Hospital Affiliated to Xiangya’ School of Medicine, Central South University, Zhuzhou, China

**Keywords:** medical image segmentation, multimodal feature fusion, semi-supervised learning, convolution transformer-based network, pancreatic tumor detection

## Abstract

**Background:**

Accurate and automated segmentation of pancreatic tumors from CT images via deep learning is essential for the clinical diagnosis of pancreatic cancer. However, two key challenges persist: (a) complex phenotypic variations in pancreatic morphology cause segmentation models to focus predominantly on healthy tissue over tumors, compromising tumor feature extraction and segmentation accuracy; (b) existing methods often struggle to retain fine-grained local features, leading to performance degradation in pancreas-tumor segmentation.

**Methods:**

To overcome these limitations, we propose SMF-Net (Semantic-Guided Multimodal Fusion Network), a novel multimodal medical image segmentation framework integrating a CNN-Transformer hybrid encoder. The framework incorporates AMBERT, a progressive feature extraction module, and the Multimodal Token Transformer (MTT) to fuse visual and semantic features for enhanced tumor localization. Additionally, The Multimodal Enhanced Attention Module (MEAM) further improves the retention of local discriminative features. To address multimodal data scarcity, we adopt a semi-supervised learning paradigm based on a Dual-Adversarial-Student Network (DAS-Net). Furthermore, in collaboration with Zhuzhou Central Hospital, we constructed the Multimodal Pancreatic Tumor Dataset (MPTD).

**Results:**

The experimental results on the MPTD indicate that our model achieved Dice scores of 79.25% and 64.21% for pancreas and tumor segmentation, respectively, showing improvements of 2.24% and 4.18% over the original model. Furthermore, the model outperformed existing state-of-the-art methods on the QaTa-COVID-19 and MosMedData lung infection segmentation datasets in terms of average Dice scores, demonstrating its strong generalization ability.

**Conclusion:**

The experimental results demonstrate that SMF-Net delivers accurate segmentation of both pancreatic, tumor and pulmonary regions, highlighting its strong potential for real-world clinical applications.

## 1 Introduction

Pancreatic cancer is projected to surpass colorectal cancer by 2040, becoming the second leading cause of cancer-related deaths after lung cancer, with a mere 12% five-year survival rate expected (1). Largely asymptomatic nature or vague symptoms, and the lack of early diagnostic biomarkers lead to the late detection of the disease when it gotten worse, even the high fatality rate of pancreatic cancer (2, 3). This emphasizes the urgent need for new therapeutic strategies to benefit the majority of patients. However, manual labeling of complex abdominal computed tomography (CT) images is time-consuming and prone to overlooking small lesions.

To address these challenges, P-MoLE, a personalized federated learning approach, enables collaborative model training across institutions without sharing sensitive data, enhancing diagnostic performance while preserving privacy (4) is highlights the promise of AI-assisted medical image segmentation in improving early detection and diagnosis of pancreatic cancer.

Therefore, developing an accurate and efficient medical segmentation model, which can reduce time and reliance on medical expertise, as well as make diagnosis faster and more accurate, is crucial for computer-aided diagnosis (5). However, due to the low contrast of pancreatic tissue in CT images and high inter-individual variability, segmentation accuracy remains limited. The rapid advancement of deep learning has marked a transformative era in medical image analysis. Medical image segmentation, as a pivotal technology, has garnered growing interest. Its primary objective is to precisely delineate anatomical structures in images, which is critical for disease diagnosis, treatment planning, and subsequent research.

Since 2012, numerous deep learning-based segmentation algorithms have been developed, including AlexNet (6), VGG-Net (7), GoogleNet (8), ResNet (9), DenseNet (10), FCNN (11), and U-Net (12). Nevertheless, accurate segmentation of pancreatic tumors and organs remains challenging due to: (a) the significant variability in pancreatic and tumor phenotypes and distribution among patients, (b) poor tumor-to-pancreas and tumor-to-background contrast, and (c) the typically small size and deep-seated location of most tumors within the pancreatic region.

**Figure 1:**
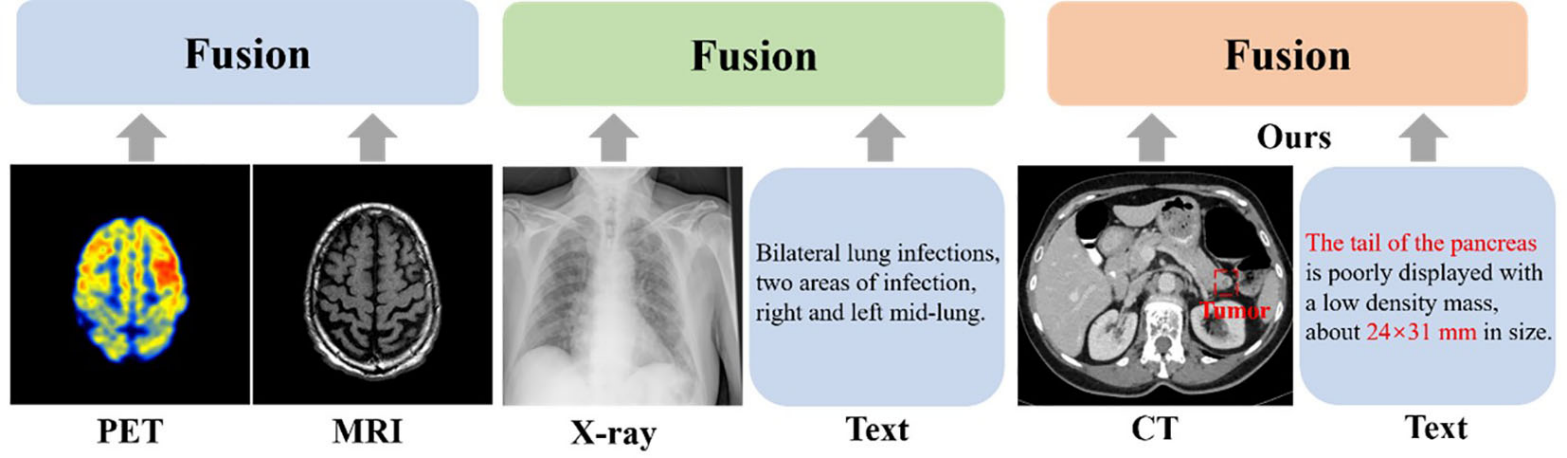
Existing multimodal fusion methods.

Recently, multimodal segmentation algorithms have emerged as a promising solution. Unlike single-modal approaches, these methods employ two or more input modalities to enhance segmentation performance. In medical imaging, multimodal segmentation includes image-to-image fusion (e.g., CT-MRI integration to extract complementary features) and image-to-text fusion (e.g., incorporating radiological annotations to augment feature learning), as depicted in **Figure 1**.

In multimodal segmentation research, Radford et al. (2021) introduced CLIP (13), which reformulates image-text matching as a pixel-level text alignment task. By leveraging pixel-text score maps to guide dense prediction models, CLIP achieves significant improvements over single-modal segmentation. However, its reliance on contrastive learning with 4 million pixel-text pairs leads to substantial computational and data demands, hindering practical deployment. To mitigate this, Zhao Yang et al. proposed ViLT (14), replacing CLIP’s visual encoder with a convolution-free architecture. This modification drastically reduces computational costs while preserving performance, offering a more efficient framework for vision-language model deployment. Subsequent studies further advanced image-text alignment techniques. For instance, Dandan Shan et al. developed C2FVL (15), incorporating text annotations with lesion counts and spatial descriptors to refine visual feature alignment, enabling precise COVID-19 lung segmentation.

Despite these advances, image-text segmentation networks often struggle to fully exploit cross-modal complementary features due to inadequate modeling and insufficient attention mechanisms, limiting their ability to retain fine-grained local representations.

To tackle this challenge, Li et al. designed LVIT (16), a hybrid CNN-Transformer architecture that integrates image and text features via a ViT fusion module while preserving local structures through the PLAM attention mechanism. Similarly, Fuying Wang et al. proposed MGCA (17), employing hierarchical alignment to progressively match visual and linguistic features across semantic scales. Their bidirectional cross-attention strategy further enhances multi-granularity token matching, optimized via contrastive learning. Rahman et al. introduced the Medical Image Segmentation Transformer (MIST), which enhances local and global feature modeling by integrating a convolutional attention mixing (CAM) decoder into a hierarchical transformer framework (18). More recently, the TAV model introduces a triguided attention module to capture visual and textual correlations across modalities, achieving 2–7% performance gains (19). An attention gate further refines feature fusion by suppressing redundancy. Additional approaches, such as CDDFuse (20) and ConVIRT (21), have explored alternative strategies for robust image-text feature extraction. Nevertheless, effectively fusing multimodal representations remains an open challenge in the field.

In addition to the challenge of effectively fusing image and text features, another critical limitation is the scarcity of high-quality multimodal (image-text) medical datasets. Currently available public multimodal medical datasets remain extremely limited, presenting significant challenges for training deep learning models. Furthermore, developing custom-built multimodal datasets poses considerable difficulties, as this process requires not only expert annotation of medical images but also the generation and precise alignment of corresponding textual descriptions - an inherently labor-intensive task. Consequently, the efficient creation and utilization of multimodal datasets has emerged as a pressing research priority (22).

While most current research focuses on pancreatic organ segmentation, few studies address the joint segmentation of pancreas and tumors. This research gap stems from two key challenges: (a) Pancreatic tumors are typically embedded in or near the pancreas, showing similar contrast to both pancreatic and surrounding tissues, making them difficult to identify accurately; (b) Tumors exhibit substantial inter-patient variability in both phenotypic characteristics and spatial distribution patterns. To confront these challenges, Pan and Bi et al. (23) developed a dynamic instance weighting approach that selectively emphasizes complex tumor instances based on guidance from simpler cases, thereby effectively transferring learned features between different complexity levels. Meanwhile, Li and Liu et al. (24) proposed a temperature-guided framework comprising three key components: balanced temperature loss, rigid temperature optimization, and soft temperature indication. This system dynamically adjusts the learning focus between tumor and pancreatic features, maintaining segmentation accuracy for healthy pancreatic tissue while improving tumor delineation.

To overcome these limitations, we present SMF-Net: a dual-path U-Net architecture integrating a dual-learner adversarial framework to enable precise segmentation of pancreatic tumors in CT imaging. The proposed network comprises two complementary branches (1): a U-shaped convolutional neural network (CNN) pathway that processes visual inputs and generates segmentation outputs, and (2) a U-shaped multimodal transformer (MTT) branch that performs cross-modal feature fusion. The MTT module, which is designed to interface with AMBERT (25), employs noise suppression while leveraging inter-modal semantic relationships to enhance textual feature extraction from AMBERT. Our architecture exhibits strong compatibility with both visual and textual features thanks to the strategically positioned Multimodal Enhanced Attention Module (MEAM) at the CNN skip connections. These MEAM units enable balanced feature representation across modalities while preserving critical anatomical details.

Additionally, we implement a semi-supervised learning paradigm to optimize resource utilization during training while enhancing model generalizability. Our Dual-learner Adversarial Network (DAS-Net) synergizes consistency regularization with adversarial training objectives. To mitigate data scarcity, a clinically annotated multimodal dataset of pancreatic tumors was compiled through collaboration with radiologists at Zhuzhou Central Hospital, containing paired CT scans and diagnostic reports. These contributions collectively advance multimodal medical segmentation research while delivering practical clinical solutions.

The main contributions of this work are as follows:

- SMF-Net architecture: We propose a novel CNN-Transformer hybrid architecture for multimodal segmentation called SMF-Net, which integrates an AMBERT text encoder to extract multi-scale textual features. The incorporated Multimodal Transformer (MTT) module enhances cross-modal feature extraction, while our Multimodal Enhanced Attention Module (MEAM) effectively preserves complementary image and text information. This design enables the comprehensive learning of pancreatic anatomical boundaries and more accurate tumor localization.
- DAS-Net framework: We have developed DAS-Net (Dual Adversarial Student Network), a semi-supervised, dual-learner, adversarial framework that integrates stability-constrained consistency regularization and discriminator-guided adversarial self-training synergistically. This unique combination significantly improves the utilization of unlabeled data during model training.
- Dataset construction: Due to the specialized knowledge required for medical image annotation, the cost of obtaining data labels is high and the amount of data is often limited. To enrich the training data, we collaborated with Zhuzhou Central Hospital to create a dataset comprising CT images of 86 pancreatic cancer patients alongside the relevant textual data.
- Comprehensive evaluation: Extensive validation on our custom-collected, multimodal pancreatic tumor dataset demonstrates state-of-the-art segmentation performance. Cross-dataset evaluations on QaTa-COV19 and MosMedData further confirm the model’s strong generalization capabilities across different imaging protocols and disease manifestations.

## 2 Related work

In this section, we examine three key methodological components: text-image feature fusion approaches, attention mechanisms, and semi-supervised learning techniques. First, we discuss the significance of text-image feature fusion in medical image segmentation and review existing methods, followed by the presentation of our proposed multimodal feature fusion module with its functional benefits. Subsequently, we analyze the critical role of attention mechanisms in multimodal data processing and introduce a novel cross-modal multi-enhanced attention mechanism. Finally, we investigate semi-supervised learning applications in medical image segmentation, with particular emphasis on our dual-student adversarial network framework.

Text-image feature fusion methods: Multimodal feature fusion represents a prominent research direction in multimodal information processing. Distinct modalities exhibit different representation characteristics, and naive fusion approaches may introduce information redundancy. Effective fusion strategies can significantly enrich feature representations. Current computer vision applications, including image captioning and segmentation, extensively employ such techniques.

Early research primarily relied on basic fusion operations such as Hadamard product, element-wise addition, or simple concatenation of heterogeneous features. While computationally simple, these methods lack theoretical sophistication. Subsequent advances have produced more sophisticated fusion paradigms, including feature-level (26), decision-level (27), hybrid-level (28), and model-level fusion (29), which constitute current state-of-the-art approaches. Our framework employs feature-level (early) fusion, which provides complementary semantic information while preserving original image characteristics with computational efficiency (30). However, early fusion may propagate noise and artifacts. To address this, we developed a Multimodal Text-Transformer (MTT) module specifically designed for compatibility with the AMBER language model. The MTT module effectively extracts textual features while suppressing noise contamination and optimally leveraging cross-modal semantic relationships.

Attention Mechanisms: Originally inspired by human cognitive processes, attention mechanisms have become fundamental components in deep learning architectures for adaptive feature selection. Bahdanau et al. first formalized attention mechanisms for neural machine translation in 2014 (31), with subsequent adaptation to computer vision by Wang et al. (32). The Transformer architecture (33) represents a landmark implementation using exclusively attention-based computations. Current research has diversified attention mechanisms into several variants, including standard attention, self-attention, and cross-attention (34–40). However, existing multi-scale attention approaches (41, 42) frequently exhibit scale-specific bias, neglecting complementary information across different scales. Our proposed Multi-modal Enhanced Attention Mechanism (MEAM) addresses this limitation by preserving fine-grained local features while effectively integrating multi-scale representations.

Semi-supervised Learning Approaches: As a well-established paradigm in machine learning, semi-supervised learning has gained renewed interest in medical image segmentation due to its ability to leverage both labeled and unlabeled data (43). Contemporary methods fall into two principal categories: regularization-based approaches and pseudo-labeling techniques. The former employs unlabeled data through consistency constraints, adversarial training, co-training paradigms, or entropy minimization, with consistency regularization demonstrating particular promise in medical imaging applications (44). The latter generates pseudo-labels from model predictions on unlabeled data, subsequently incorporating them into the training set, that has shown empirical success across various segmentation tasks (45–48).

Both paradigms present inherent limitations. Regularization-based methods require careful design of data augmentation strategies to produce meaningful sample variations; excessive perturbations may degrade model performance, while insufficient variations yield ineffective regularization. Pseudo-labeling methods risk error propagation when incorrect predictions are treated as ground truth during training. Furthermore, divergent perturbations across co-trained sub-networks may induce prediction inconsistencies, exacerbating pseudo-label uncertainty. To mitigate these issues, we extend the dual-student framework (49) by incorporating an attention-equipped discriminator network, proposing the Dual-Adversarial-Student Network (DAS-Net) architecture.

Despite the significant progress made in multimodal medical image segmentation, existing methods still face key challenges. Traditional text-image fusion often leads to redundant or weakly aligned features, attention mechanisms may suffer from scale bias and incomplete cross-modal interaction, and semi-supervised learning approaches are vulnerable to pseudo-label noise and instability from inconsistent augmentations. Our framework addresses these challenges by proposing the MTT module for robust noise-suppressed fusion, the MEAM attention mechanism for effective multi-scale integration, and the DAS-Net framework to stabilize semi-supervised learning with dual-student co-training enhanced by an attention-based discriminator. These contributions collectively improve feature representation quality, learning stability, and segmentation accuracy beyond existing approaches.

## 3 Materials and methods

### 3.1 Data collection

**Figure 2:**
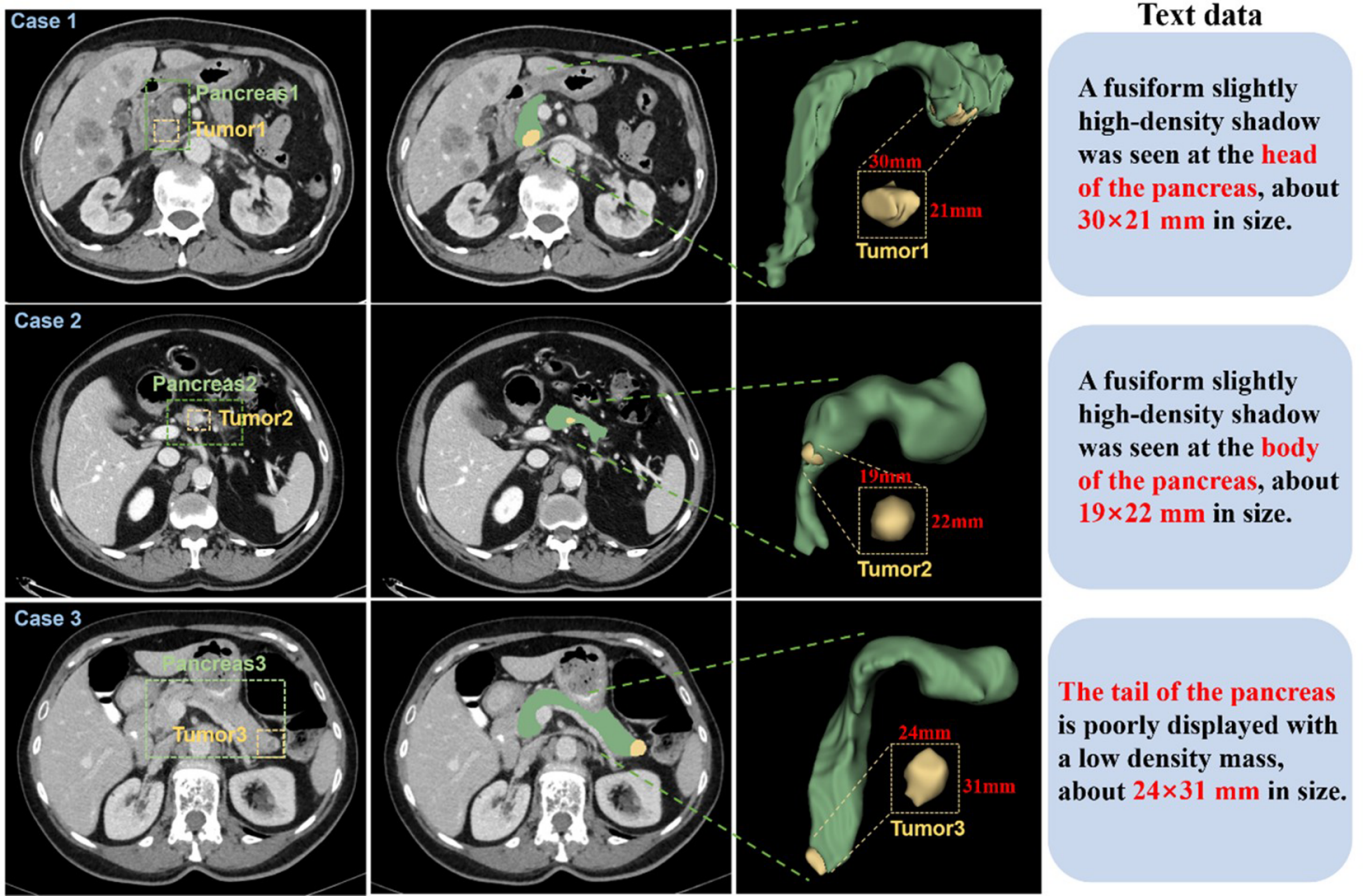
CT images of 3 patients were selected from the self-constructed dataset. Each case displays (left to right): three magnified axial slices, 3D reconstruction, and associated clinical annotations. Pancreatic anatomy is demarcated by green dashed boundaries, with yellow dashed contours highlighting tumor regions.

Abdominal CT imaging reveals considerable anatomical variability, with substantial inter-image differences in structural morphology, dimensional characteristics, and tissue density. Abdominal CT imaging presents considerable anatomical complexity, with substantial variations in structural morphology, dimensional characteristics, and tissue density across patients (**Figure 2**). To establish a comprehensive multimodal pancreatic tumor dataset, we selected three representative cases from our institutional collection, demonstrating tumors in distinct anatomical locations: the pancreatic head (Case 1), body (Case 2), and tail (Case 3). The pancreatic body lesion (Case 2) proved particularly challenging for detection, exhibiting both small tumor volume (mean diameter nearly 2 cm) and low contrast enhancement.

The improved dataset comprises two parts: (a). A filtered dataset from the Medical Segmentation Decathlon (MSD) (50), including 235 sets of CT images with pancreatic and tumor labels from patients, after excluding 47 sets of duplicate or unclear background segmented data. This dataset is provided by Memorial Sloan Kettering Cancer Center (New York, NY, USA) and poses a challenge due to the imbalance in labeling small pancreatic tumor structures within a large background. (b). Original CT images in DICOM format from 108 pancreatic cancer patients, including non-contrast, arterial, and venous phases, provided by Zhuzhou Central Hospital. Under radiologist supervision, we implemented rigorous quality control to exclude non-diagnostic images based on the following criteria: a) duplicate examinations, b) excessive motion artifacts (n=14), c) inadequate spatial resolution (n=8), retaining 86 qualifying cases. All pancreatic lesions were manually segmented using 3D-Slicer, with tumor dimensions measured across three orthogonal planes. The resulting annotations and quantitative measurements (maximum diameter, volume) were systematically recorded in standardized metadata files. All annotations were verified by radiologists. Ultimately, this study’s pancreatic tumor medical dataset contains 321 CT images of pancreatic cancer patients with pancreas and tumor annotations. In the experiment, 35 randomly selected CT images from the 321 sets were used as the test set to evaluate model performance, with the remaining sets used as the training set.

The segmentation targets of this paper are pancreatic organs and pancreatic tumors. Due to the pancreas’s complex anatomical position, tumors significantly impact surrounding organs (51). Based on the location characteristics of tumors in the annotated patient CT images, we classified them into four types:(a). Pancreatic head cancer, where the tumor is located in the head of the pancreas, usually appearing as a localized mass or enlargement in the pancreatic head region on CT images. (b). Tumor located in the body and tail of the pancreas, usually showing lower contrast compared to surrounding tissues and probably causing local enlargement of the pancreas. (c). Tumor located in the tail of the pancreas without spreading, small in size with uneven density, depending on the tissue composition and necrosis degree of the tumor. (d). Tumor located in the tail of the pancreas with spreading, large lesion area, appearing as a localized high-density area on CT images.

### 3.2 Deep learning method

To overcome the segmentation challenges posed by the small size, irregular shape, and complex spatial distribution of pancreatic tumors, we propose SMF-Net, a novel framework for accurate pancreas and tumor segmentation in medical images. We employ a hybrid CNN-Transformer encoder as the backbone network for feature extraction and introduce the MEAM (Multi-modal enhanced attention mechanism) to integrate high-level semantic features with low-level fine-grained spatial features. This fusion mechanism establishes long-range dependencies, improves feature discriminability, and enables effective cross-scale feature fusion

#### 3.2.1 U-MTT Branch

**Figure 3:**
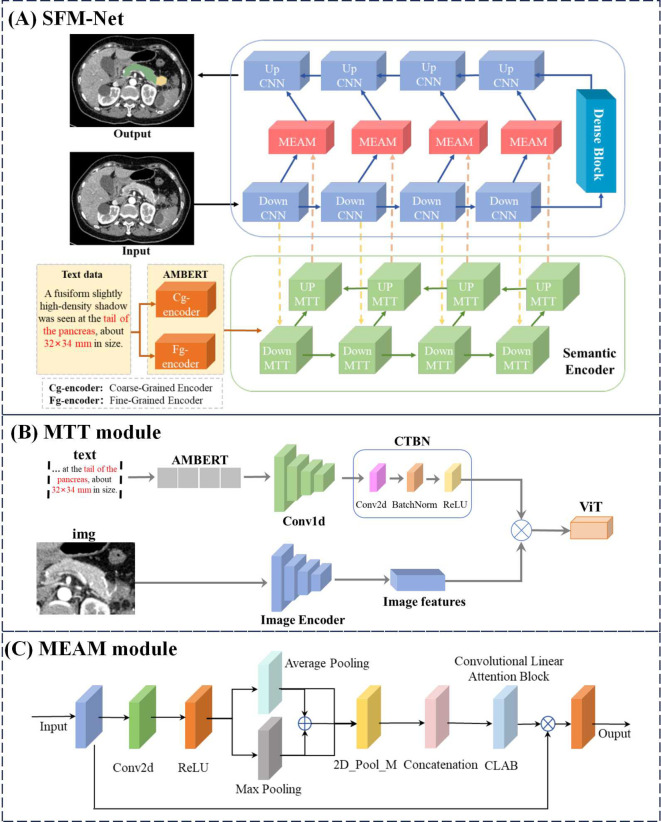
Overall network structure of SFM-Net.


As illustrated in **Figure 3A**, our backbone architecture comprises two U-shaped networks combining CNNs and Transformers, where the U-shaped Transformer (52) represents our proposed MTT module. This U-shaped MTT module is specifically designed for multimodal feature fusion between text and image representations. The module initially processes text embeddings from the AMBERT pre-trained language model, which have undergone both fine-grained (Fg-encoder) and coarse-grained (Cg-encoder) encoding. The fusion process can be formally expressed as shown in Equations 1–3:

(1) $$\begin{array}{c} x{\;}_{\text{text}}=Y{\;}_{\text{AMBERT}}\left({\text{input }}_{\text{text}}\right) \end{array}$$

(2) $$\begin{array}{c} x{\;}_{\text{img}}=Y{\;}_{\text{DownCNN},1}\left({\text{input }}_{\text{img}}\right) \end{array}$$

(3) $$\begin{array}{c} Y{\;}_{\text{DownMTT}}=MTT\left({\text{x }}_{\text{img}},{\text{ x }}_{\text{text}}\right) \end{array}$$

here $input_{img}$ and $input_{text}$ denote the input image and text data streams respectively, $x_{img}$ corresponds to the features extracted by the first Down CNN layer, $x_{text}$ represents the text features encoded by AMBERT, and $Y_{DownMTT}$ indicates the fused features generated by the MTT module. Architecturally, each MTT module maintains the standard transformer encoder configuration, containing multi-head self-attention mechanisms and MLP layers, along with conventional convolutional operations and activation functions. In the subsequent processing stages, each Down MTT layer (i ∈ {1,2,3}) incorporates both the hierarchical features from the preceding Down MTT module and the corresponding feature maps from the parallel Down CNN pathway are formulated as shown in Equation 4:


(4) $$\begin{array}{c} Y{\;}_{\text{DownATT},\text{i}+1}=MTT\left({\text{Y }}_{\text{DownMTT},\text{i}}+{\text{x }}_{\text{img},\text{i}+1}\right) \end{array}$$

The processed features are then propagated through Up MTT modules to the MEAM component, where they undergo integration with the corresponding Down CNN features before being processed by the Up CNN modules.

#### 3.2.2 U-CNN branch

**Figure 3A** illustrates the U-shaped CNN branch responsible for processing image inputs and generating the final segmentation output. Each CNN module incorporates sequential Conv, BatchNorm (BN), and ReLU activation operations. Between consecutive DownCNN modules, MaxPool layers perform feature downsampling. The transformation at each DownCNN level is defined as shown in Equations 5, 6:


(5) $$\begin{array}{c} Y{\;}_{\text{DownCNN},1}=Y{\;}_{\text{DownCNN}}\left({\text{input }}_{\text{img}}\right) \end{array}$$

(6) $$\begin{array}{c} Y{\;}_{\text{DownCNN},\text{i}+1}=MaxPool\left({\text{Y }}_{\text{DownCNN},\text{i}}\right) \end{array}$$

where $Y_{DownCNN}$ denotes the input to the i-th DownCNN module, which undergoes processing through both the CNN operations and MaxPool downsampling to produce $Y_{DownCNN,i+1}$ . These hierarchical features then combine with corresponding UpMTT features through residual connections, creating cross-modal representations. To maintain balanced contribution from both modalities while preserving critical feature information for segmentation accuracy, we introduce the MEAM module. The integrated features subsequently propagate through the MEAM-enhanced pathway, progressively upsampling via UpCNN modules to yield the final segmentation output.

#### 3.2.3 Match AMBERT MTT

**Figure 3B** presents the AMBERT-aligned MTT fusion module, designed to enhance text feature extraction from AMBERT while capturing cross-modal semantic relationships between textual and visual information. This fusion mechanism effectively leverages inter-modal feature interactions to boost performance. The processing pipeline first transforms input text through AMBERT’s dual-path extraction, obtaining both coarse-grained and fine-grained textual representations ( $Y_{AMBERT}$ ). These text features then undergo transformation via our custom CTBN layer - a sequential combination of Conv2d, BatchNorm, and ReLU operations - before being combined with image features ( $X_{img}$ ) via element-wise multiplication. The integrated features are further processed by a Vision Transformer (ViT) (53) module to produce the final output ( $Y_{MTT}$ ). The complete transformation can be formally expressed as shown in Equation 7:


(7) $$\begin{array}{c} Y{\;}_{\text{MTT}}=Y{\;}_{\text{ViT}}\left[\prod_{\text{x}}{\text{X }}_{\text{img}}⊗{\text{Y }}_{\text{CBTN}}\left({\text{Y }}_{\text{AMBERT}}\left({\text{X }}_{\text{text}}\right)\right)]\right] \end{array}$$

where $X_{img}$ and $X_{text}$ denote the image and text inputs respectively, $Y_{AMBERT}$ represents AMBERT’s hierarchical text features, $Y_{CBTN}$ and $Y_{ViT}$ correspond to the CTBN and ViT transformation operations. The MTT fusion module generates more comprehensive text representations compared to conventional multimodal fusion approaches, demonstrating superior capability in modeling text-image semantic relationships.

#### 3.2.4 Multi-modal enhanced attention mechanism

**Figure 3C** illustrates the MEAM module, which maintains balanced consideration of both modality inputs while preserving original feature representations. Drawing inspiration from CBAM (54) mean-maximum fusion strategy, MEAM employs parallel pooling pathways. The processing begins with 2D convolutional transformation followed by nonlinear activation, after which features undergo parallel Average Pooling (AP) and Max Pooling (MP) operations. These pooled features then undergo additional 2D max pooling (P_M) for salient feature extraction. The processed AP and MP features are then concatenated with residual connections, followed by another 2D max pooling operation. These three processed feature streams are then integrated via residual concatenation to preserve original feature characteristics. Incorporating concepts from CLAB (55), we implement a simplified CLAB layer for final feature alignment. The aligned features are then combined with original inputs through element-wise multiplication to retain critical feature information. The complete MEAM operation can be formally expressed as shown in Equation 8:


(8) $$\begin{array}{c} Y{\;}_{\text{MEAM}}=\prod_{\text{x}}\text{X}⊗{\text{Y }}_{\text{CLAB}}\left[{\text{AP }}_{{\text{P}}_{\text{M}}}+{\text{MP }}_{{\text{P}}_{\text{M}}}+\left(\text{AP}+\text{MP}\right){\;}_{{\text{P}}_{\text{M}}}\right] \end{array}$$

where X denotes input features, AP and MP represent average- and max-pooled features respectively, $P_{M}$ indicates 2D max pooling, $Y_{CLAB}$ corresponds to the feature alignment transformation, and Y represents the final output. Through this architecture, MEAM effectively combines multi-scale pooling operations with residual connections and feature recombination to maximize information utilization from original inputs.

#### 3.2.5 Semi-supervised dual-student adversarial learning method

**Figure 4:**
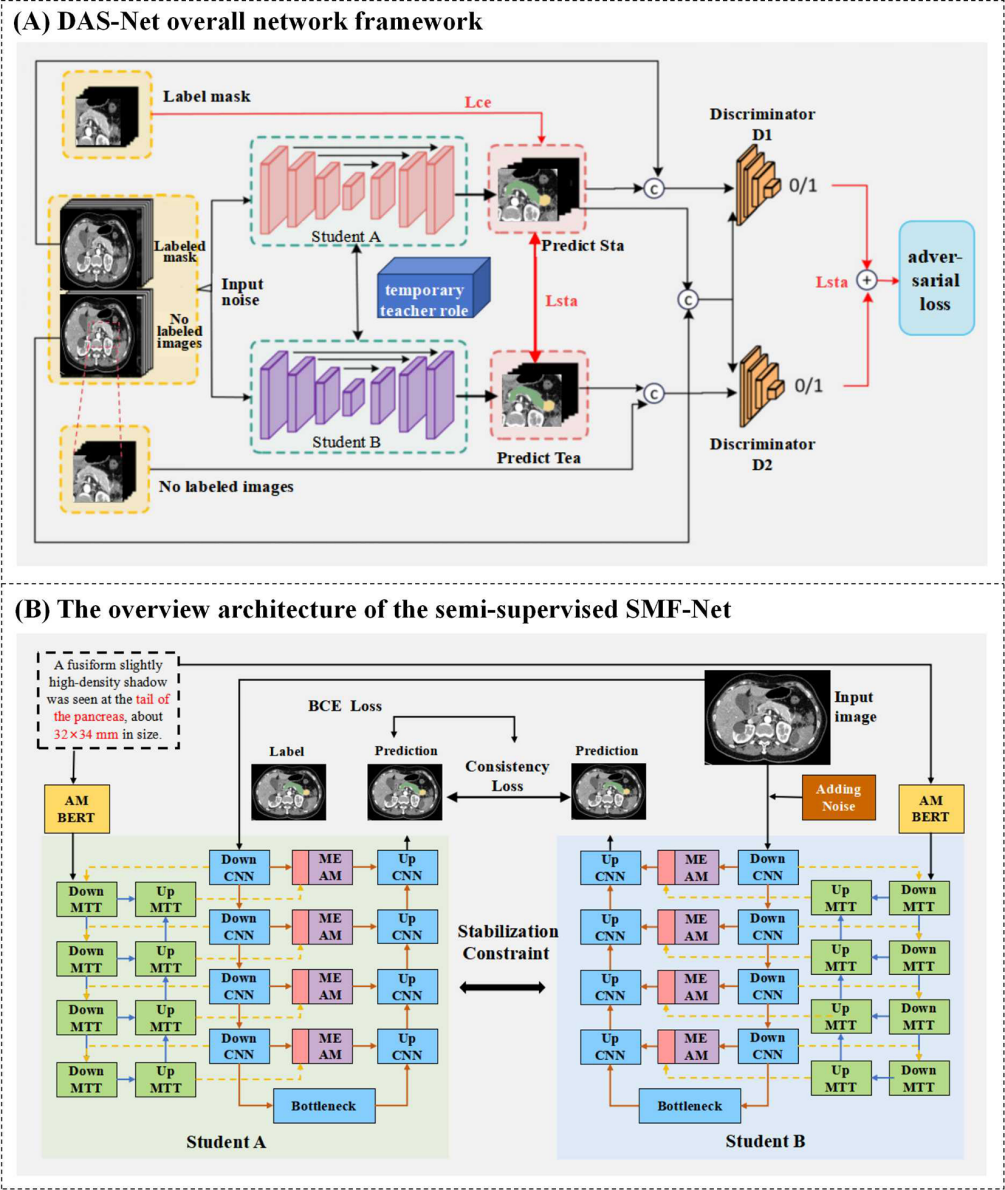
**(A)** DAS-Net overall network framework. **(B)** The overview architecture of the semi-supervised SMF-Net.

As previously discussed, semi-supervised learning approaches are crucial for mitigating data scarcity challenges in medical image segmentation. Illustrated in **Figure 4A**, our proposed Dual Adversarial Student Network (DAS-Net) addresses the limited availability of annotated multimodal medical data by effectively leveraging both scarce labeled samples and abundant unlabeled data to enhance segmentation accuracy.

The architecture incorporates two structurally identical discriminator networks within the dual-learner framework. Discriminator D operates on reliable pseudo-labels generated from unlabeled data during self-training, enabling robust quality assessment of predictions across both labeled and unlabeled samples. The adversarial training paradigm alternates between generators and discriminators, progressively improving the segmentation network’s ability to produce high-confidence predictions (approaching unity) for unlabeled data. The resulting segmentation objective function is formulated as shown in Equation 9:


(9) $$\begin{array}{c} L{\left(\theta \right)}_{S}=L_{S}\left({\overset{\wedge }{y}}_{i},y_{i}\right)+\lambda \left(\begin{array}{c} L_{semi}\left({\overset{\wedge }{y}}_{u},{\overset{\wedge }{y}}_{ema}\right)+L_{adv1}\left(D_{1}\left(x_{u},{\overset{\wedge }{y}}_{u}\right),\;1\right)\\ +L_{adv2}\left(D_{2}\left(x_{u},{\overset{\wedge }{y}}_{u}\right),1\right) \end{array}\right) \end{array}$$

The objective functions of discriminators D1​ and D2​ can be defined as shown in Equations 10, 11:


(10) $$\begin{array}{c} L{\left(\theta \right)}_{D_{1}}=L_{adv1}\left(D_{1}\left(x_{i},{\overset{\wedge }{y}}_{i}\right),\;1\right)+L_{adv1}\left(D_{1}\left(x_{u},{\overset{\wedge }{y}}_{u}\right),0\right) \end{array}$$

(11) $$\begin{array}{c} L{\left(\theta \right)}_{D_{2}}=L_{adv2}\left(D_{2}\left(x_{ema},{\overset{\wedge }{y}}_{ema}\right),\;1\right)+L_{adv2}\left(D_{2}\left(x_{u},{\overset{\wedge }{y}}_{u}\right),0\right) \end{array}$$

Where $L_{s}$ is the Dice loss, $L_{semi}$ ​ is the Mean Squared Error (MSE) loss, $L_{adv1}$ ​ and $L_{adv2}$ ​ are multi-class cross-entropy losses. $x_{i}$ and $y_{i}$ ​ correspond to the input image training data and its true labels, while $x_{u}$ ​ and $x_{ema}$ ​ correspond to the input unlabeled data and noise perturbations. ${\overset{\wedge }{y}}_{u}$ ​ and ${\overset{\wedge }{y}}_{i}$ ​ are the segmentation prediction results for labeled and unlabeled data, respectively, and ${\overset{\wedge }{y}}_{ema}$ is the segmentation prediction result from the teacher model under EMA weight propagation. The weighting coefficient is defined in a gradually increasing Gaussian curve manner according to reference (56), and can be expressed as shown in Equation 12:


(12) $$\begin{array}{c} \lambda =\delta \;e^{\left(-5{\left(1-I\right)}^{2}\right)} \end{array}$$

Where I is the number of training epochs for the model. In the training results of the mean teacher method, the weight parameters of the teacher model are the EMA accumulation of the student model parameters (57), which can be defined as shown in Equation 13:


(13) $$\begin{array}{c} {\theta^{'}}_{t}=\alpha {\theta^{'}}_{t-1}+\left(1-\alpha \right)\theta_{t} \end{array}$$

Where ${\text{θ}}_{t}^{'}$ ​ represents the parameters to be updated for the teacher model, ${\text{θ}}_{t}$ ​ is the weight parameters of the student model, and α is the hyperparameter for the smoothing coefficient. The value of α determines the dependency relationship between the teacher and student models. According to references (58) and practical experiments, the best performance is achieved when α =0.999.

The proposed Dual Adversarial Student Network (DAS-Net) framework initializes two architecturally identical yet independently trained student models with synchronized parameters. During training, each branch maintains its own weight updates while exchanging learned feature representations through a shared information channel. To maintain prediction consistency between branches and ensure training stability, we impose regularization constraints on the unlabeled data processing pipeline. The framework processes labeled data through supervised loss computation while simultaneously extracting valuable feature representations from unlabeled images via consistency constraints. These mechanisms are further enhanced through adversarial learning components.

The comprehensive loss function for DAS-Net combines weighted contributions from both student networks (Student A and Student B), each comprising three key components: supervised loss, unsupervised consistency loss, and adversarial loss. Both branches share identical loss formulations, with Student A’s total loss $L^{a}$ expressed as shown in Equation 14:


(14) $$\begin{array}{c} L^{a}=L_{seg}^{a}+\lambda_{1}\cdot L_{cons}^{a}+\lambda_{2}\cdot L_{sta}^{a}+\lambda_{3}\cdot L_{adv}^{a} \end{array}$$

Where $L_{seg}$ ​ represents the supervised loss, which includes the cross-entropy loss and the Dice loss. $L_{cons}$ ​ denotes the loss function for the consistency constraint. $L_{sta}$ represents the stabilization constraint loss for Student A, and $L_{adv}$ ​ expresses the adversarial loss for Student A. The parameters $\lambda_{1}$ , $\lambda_{2}$ , and $\lambda_{3}$ are the weighting coefficients used to balance the constraints. (See Section 4.4.3 for specific experiments).

Consistency Loss ( $L_{cons\;}^{a},\lambda_{1}$ **):** This term minimizes prediction discrepancies for the same unlabeled sample under different perturbations (e.g., Gaussian noise, rotation). However, excessively high λ_1_ values can induce oversensitivity to perturbations, reducing model robustness (59).

Stabilization Loss ( $L_{sta\;}^{a},\lambda_{2}$ **):** This selectively applies consistency constraints exclusively to high-confidence pseudo-labels. Setting λ_2_ too high with low confidence thresholds introduces label noise, whereas overstringent thresholds reduce valid samples for learning.

Adversarial Loss ( $L_{adv\;}^{a},\lambda_{3}$ ): A discriminator aligns feature distributions between labeled and unlabeled data. Due to the inherent instability of adversarial training convergence, this loss typically requires significantly lower weighting (empirically $\lambda_{3}$ < 0.1) compared to other components.

The supervised loss function can be expressed as shown in Equation 15:


(15) $$\begin{array}{c} L_{\text{seg}}=\frac{L_{CE}\left(x_{i},y_{i}\right)+DICE\left(x_{i},y_{i}\right)}{2} \end{array}$$

Where the $L_{seg}$ ​ loss function combines the cross-entropy loss $L_{CE}$ and the Dice similarity coefficient to simultaneously focus on both positive and negative samples. The formula for the cross-entropy loss function in the supervised loss is as shown in Equation 16:


(16) $$L_{CE}^{a}=-\sum_{i,j,c}y_{l_{\left(i,j,c\right)}}\log S_{a}{\left(x_{l}\right)}_{\left(i,j,c\right)}$$

For the unlabeled images, the segmentation network Student A is applied twice to generate two prediction results, $SaT\left(x_{u}\right)$ and $T\left(Sax_{u}\right)$ . The pixel-level consistency error $\epsilon^{a}\in R^{h\times w}$ can be calculated, which is used to compute the consistency and stabilization losses as shown in Equation 17:


(17) $$\begin{array}{c} \epsilon^{a}{\left(x_{u}\right)}_{\left(i,j\right)}={\left(S_{a}{\left(T\left(x_{u}\right)\right)}_{\left(i,j\right)}-T{\left(S_{a}\left(x_{u}\right)\right)}_{\left(i,j\right)}\right)}^{2} \end{array}$$

Where $S_{a}$ represents one of the student networks, and $x_{u}$ ​ denotes the input unlabeled data. The Euclidean distance is used to measure the consistency of the predictions, where a smaller value of $\epsilon^{a}$ indicates greater stability of the sample. To leverage the semantic information from the unlabeled data sample $x_{u}$ ​, consistency constraints are applied to each student branch. For the unlabeled image $x_{u}$ ​, segmentation networks $S_{a}$ and $S_{b}$ ​ are used to generate two segmentation results, $SaT\left(x_{u}\right)$ and $T\left(Sax_{u}\right)$ . Consequently, the formula for calculating the consistency loss function is as shown in Equation 18:


(18) $$L_{cons}^{a}=\frac{1}{h\times w}\sum_{i,j}^{h,w}\varepsilon^{a}{\left(x_{u}\right)}_{\left(i,j\right)}$$

To enhance the stability of model training, a stabilization constraint is applied to the sample when the semantic information of the input sample matches the label and the prediction confidence exceeds a predefined threshold. For the Student A branch, the formula for calculating the pixel stabilization loss is as shown in Equation 19:


(19) $$\begin{array}{c} l_{sta}^{a}\left(x\right)=\{\begin{array}{cc} {\left[\epsilon^{a}\left(x\right)<\epsilon^{b}\left(x\right)\right]}_{1}L_{mse}\left(x\right),\; & r^{a}=r^{b}=1\\ r^{a}L_{mse}\left(x\right),\; & r^{a}、r^{b}\neq 1 \end{array} \end{array}$$

Where $L_{mse}$ ​ is the Mean Squared Error used to measure the consistency between the two predicted outputs. The specific expression is as shown in Equation 20:


(20) $$\begin{array}{c} L_{mse}{\left(x_{u}\right)}_{\left(i,j\right)}={\left(S_{a}{\left(T\left(x_{u}\right)\right)}_{\left(i,j\right)}-S_{b}{\left(T\left(x_{u}\right)\right)}_{\left(i,j\right)}\right)}^{2} \end{array}$$

The overall stabilization loss function is calculated as shown in Equation 21:


(21) $$L_{sta}^{a}(x_{u})=\frac{1}{h\text{x}w}\sum_{i,j}^{h,w}l_{sta}^{a}{\left(x_{u}\right)}_{\left(i,j\right)}$$

In conclusion, we present a novel multimodal hybrid architecture that synergistically combines CNN and Transformer features within a dual U-shaped network framework, implemented through our proposed semi-supervised Dual Adversarial Student learning paradigm (**Figure 4B**). This comprehensive approach effectively addresses the fundamental challenges of limited annotated data in medical image analysis while leveraging the complementary strengths of CNN and Transformer architectures for robust multimodal segmentation.

## 4 Experiments

### 4.1 Experimental environment

To verify the performance of SMF-Net, we conducted necessary comparative experiments and ablation studies. The approach presented in this chapter is implemented using the PyTorch framework. The main server specifications are as follows: the operating system is Ubuntu 20.04.12 LTS, the CPU is an Intel(R) Xeon(R) Gold 5218, the GPU is an NVIDIA NTX4090 24G, and the memory capacity is 256GB. The training and validation sets are divided from the original training set, ranging from 10% to 50%.

During experimentation, only basic data augmentation strategies were employed, including random rotation, scaling, flipping, and brightness adjustment. To ensure fair comparison across all methods, identical input dimensions, preprocessing protocols, and training loss functions were applied to all three datasets without utilizing additional pre-training data. The Adam optimizer was used with an initial learning rate of 3e-4 for the MosMedData+ and QaTa-COV19 datasets, and 1e-3 for the MPTD dataset, while maintaining a consistent momentum of 0.99. An early stopping mechanism is employed, terminating the training if the model’s performance does not improve within 100 epochs. Additionally, considering the varying scales of the datasets, different batch sizes were configured: with input resolution fixed at 256×256, batch sizes were set to 24 for MosMedData+ and MPTD, and 16 for QaTa-COV19.

### 4.2 Loss function and evaluation index

#### 4.2.1 Dice (Dice coefficient)

The Dice coefficient measures the similarity between two samples, with values closer to 1 indicating higher similarity. In image segmentation tasks, a high Dice coefficient (e.g., 0.8 or higher) suggests good segmentation performance. It is calculated as shown in Equation 22:


(22) $$\begin{array}{c} Dice=\sum_{i=1}^{N}\sum_{j=1}^{C}\frac{1}{NC}\cdot \frac{2\left|p_{ij}\cap^{\;}y_{ij}\right|}{\left(\left|p_{ij}\right|+\left|y_{ij}\right|\right)}=1-L_{Dice} \end{array}$$

#### 4.2.2 MIoU (Mean Intersection over Union)

MIoU, or Mean Intersection over Union, is an indicator used to measure the effectiveness of medical image segmentation. It calculates the overlap between the segmentation result predicted by the model and the actual segmentation label. By computing the intersection and union of the predicted results for each category with the real labels, the proportion is determined, and the average proportion across all categories is obtained. A higher MIoU value indicates that the prediction is closer to the true value, reflecting better segmentation performance. The formula for calculating MIoU is as shown in Equation 23:


(23) $$\begin{array}{c} mIoU=\sum_{i=1}^{N}\sum_{j=1}^{C}\frac{1}{NC}\cdot \frac{\left|p_{ij}\cap^{\;}y_{ij}\right|}{\left|p_{ij}\cup^{\;}y_{ij}\right|} \end{array}$$

#### 4.2.3 95HD (95% Hausdorff distance)

The Hausdorff Distance (HD) is used to measure the distance between two subsets in a space. In the field of medical image segmentation, is it is particularly important to quantify the difference between predicted values and ground truth segmentation values. As it effectively captures the utmost discrepancy between the predicted and ground truth segmentation outcomes, HD is frequently employed for measuring the performance of models when it comes to segmenting boundary regions. Its expression is as shown in Equation 24:


(24) $$\begin{array}{c} H\left(A,B\right)=max\left\{\underset{a\in A}{max}\left(\underset{b\in B}{min}||\text{a}-\text{b}||\right),\underset{b\in B}{max}\left(\underset{a\in A}{min}||\text{b}-\text{a}||\right)\right\} \end{array}$$

Where ||·|| is the distance norm between point set A and point set B. In all experiments, the segmentation accuracy and performance of the model are assessed by DSC and HD.

#### 4.2.4 MAE (Mean Absolute Error)

When the output predicted by the segmentation model is a probability map, the MAE can evaluate the error between the predicted probabilities and the true labels. MAE provides a numerical measure of the prediction error for each pixel, reflecting the average performance of the model at the pixel level. The formula for calculating MAE is as follows, where the true label of the *i-th* pixel is denoted and the predicted probability of the *i-th* pixel is described, and n is the total number of image pixels as shown in Equation 25.


(25) $$\begin{array}{c} MAE=\frac{1}{n}\sum_{i=1}^{n}\left|y_{i}-{\left(\overset{\wedge }{y}\right)}_{i}\right| \end{array}$$

### 4.3 Contrast experiment

#### 4.3.1 Comparison with current state-of-the-art fully supervised methods

Due to the lack of public multimodal pancreatic datasets, we evaluated our model’s performance on smaller and more challenging lesions using our in-house multimodal pancreatic tumor dataset (MPTD) in comparative and ablation experiments. SMF-Net was compared against five fully supervised single-modal segmentation methods (U-Net, ATT-UNet, UNet++, TransUNet, Swin-Unet) and three multimodal methods (ViLT, LViT-NT [without text], LViT-WT [with text]). For semi-supervised experiments, we used 50% labeled and 25% unlabeled training data.

As shown in **Table 1**, under 100% label rate, SMF-Net outperformed all five single-modal baselines on MPTD. At 50% label rate, our model achieved a mean Dice score of 67.61%, surpassing UNet++ and TransUnet, demonstrating performance comparable to fully supervised single-modal methods. At full supervision, SMF-Net improved the tumor segmentation Dice score by 3.82% over Swin-Unet (the best single-modal method), validating the efficacy of text-guided feature learning.

**Table 1:** DSC and HD of the different methods based on the MPTD dataset.

Method	Label ratio (%)	Pancreas (%)	Tumor (%)	DSC (%)	95HD (Voxel)
U-Net	100	67.67	50.60	59.13	20.61
Att-UNet	100	69.81	52.74	61.27	18.02
UNet++	100	72.12	55.03	63.57	17.84
TransUNet	100	75.30	59.23	67.26	13.27
Swin-Unet	100	75.72	60.39	68.05	15.68
ViLT	100	74.52	58.19	66.36	17.82
LViT-NT	100	76.37	59.26	67.82	16.51
LViT-WT	100	77.01	60.03	68.52	14.51
**Ours**	**100**	**79.25**	**64.21**	**71.73**	**9.59**
**Ours**	**50**	76.93	58.29	67.61	11.65

The best scores are highlighted.

In multimodal comparisons (**Table 1**), SMF-Net achieved a 5.37% higher mean Dice score than ViLT and a 3.35% improvement over LViT-WT in tumor segmentation. Thus, SMF-Net consistently surpasses state-of-the-art (SOTA) methods in both single and multimodal settings.

**Figure 5:**
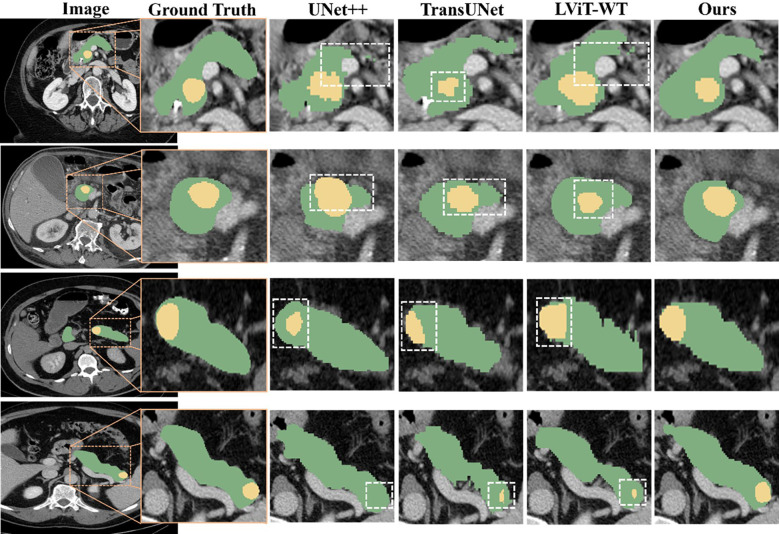
Qualitative comparison among the segmentation results obtained using UNet++, TransUNet, LViT-WT, and our model. The pancreas is marked in green, and the tumor is marked in yellow. Inaccurate segmentation areas are outlined with white dashed lines. Three distinct cases are presented, enlarged from the axial view to show more details. From top to bottom, the tumors were located in the pancreatic head, body, and tail of the pancreas.

The prediction results are presented in **Figure 5**, demonstrating the superior segmentation performance of our proposed model compared to UNet++, TransUNet and LViT-WT. Although there are some differences in the segmentation results, particularly in the shape and size of the tumors, the predicted results from the proposed model closely resemble the actual annotated results.

#### 4.3.2 Comparison of semi-supervised methods

As shown in **Table 2**, we evaluated the segmentation performance of Dual-Student-SMF-Net across varying label rates, using BERT-based LViT (60) as the baseline. Comparisons included LViT variants with (LViT-WT) and without (LViT-NT) text guidance. Results demonstrate that both our method and LViT-WT consistently surpass LViT-NT, validating the efficacy of text-enhanced segmentation.

**Table 2:** Semi-supervised experimental comparison of the different methods based on the MPTD dataset.

Method	Label ratio (%)	Pancreas (%)	Tumor (%)	DSC (%)	95HD (Voxel)
LViT-NT	25	67.85	45.19	56.52	20.63
LViT-WT	25	69.45	47.38	58.42	17.96
**Ours**	25	**71.46**	**50.72**	**61.09**	**14.12**
LViT-NT	50	72.43	54.13	63.28	18.63
LViT-WT	50	73.93	56.02	64.97	16.79
**Ours**	50	**76.93**	**58.29**	**67.61**	**11.65**

The best scores are highlighted.

**Figure 6:**
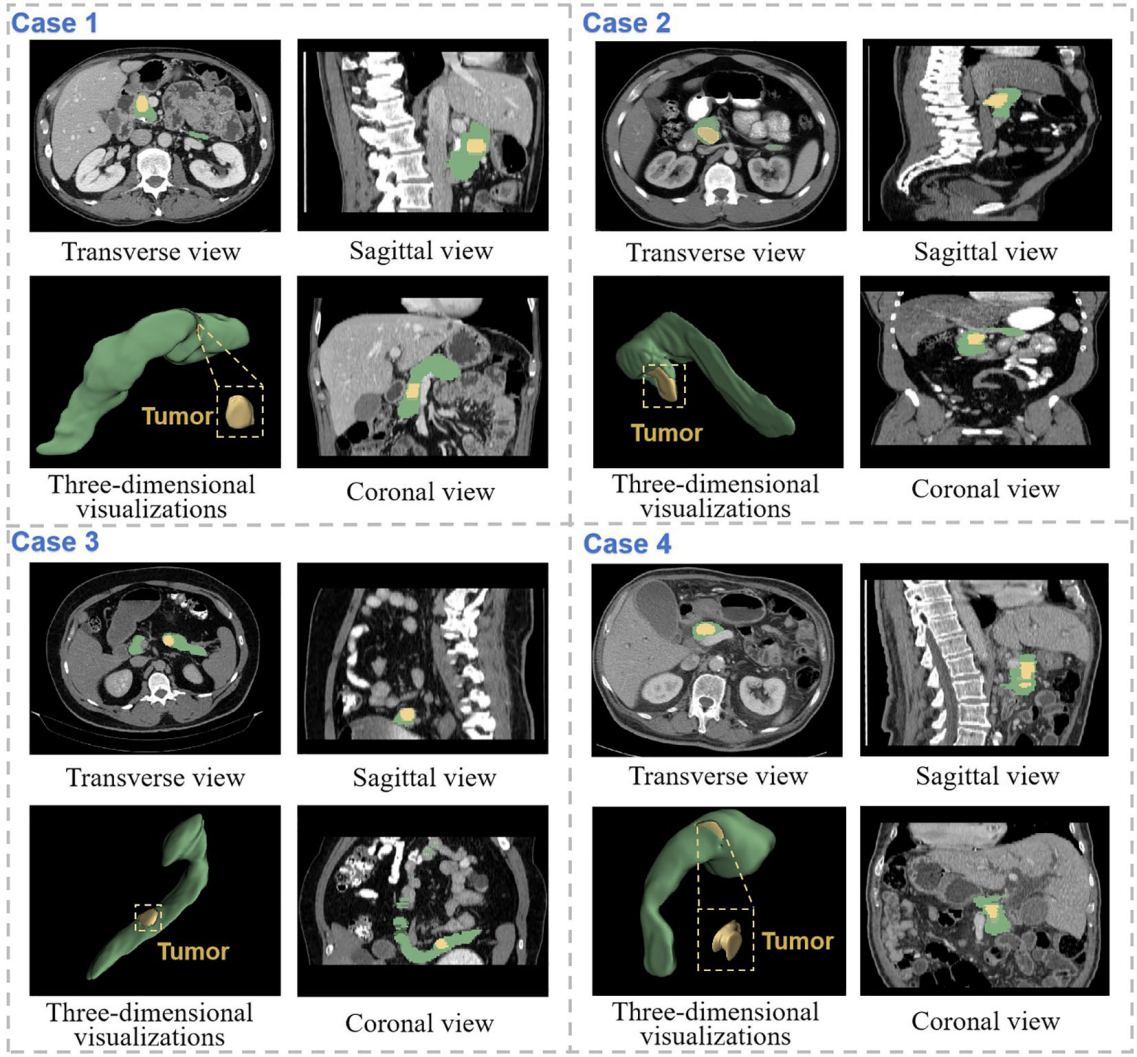
Demonstration of segmentation results of SMF-Net trained with 75% dual-student semi-supervised method. The pancreas is marked in green, and the tumor is marked in yellow. Coronal, sagittal and transverse views of the CT segmentation results of four different cases are shown separately, as well as three-dimensional visualizations.

As illustrated in **Figure 6**, we present representative segmentation results from SMF-Net under semi-supervised learning. Given the pancreas’ small size relative to other organs, where minor segmentation errors can significantly impact performance, our method demonstrates robust accuracy. Notably, the proposed Dual Adversarial Student Network (DAS-Net) enables SMF-Net to maintain excellent segmentation quality even with only 50% supervised training.

### 4.4 Ablation experiment

Our primary contributions include: (a) a Multi-granularity Text-Target fusion module (MTT) that aligns coarse-to-fine textual features, (b) a Multi-level Enhanced Attention Mechanism (MEAM) for cross-modal representation learning, and (c) their integration with a semi-supervised dual-student framework. We validate these innovations through two ablation studies on the MPTD dataset, examining: text feature extraction efficacy and MEAM component contributions.

#### 4.4.1 Text feature extraction

**Table 3** presents comparisons using BERT-based LViT as baseline. Here, LViT-NT and OUR-NT denote configurations where text features were disabled (replaced with non-informative constants). Results demonstrate that text features improved tumor Dice scores by 1.83% for LViT-WT and 3.06% for our model, confirming their segmentation-enhancing effect. Notably, our text-enabled model outperformed LViT-WT by 2.8% in Dice coefficient, establishing its superior segmentation capability.

**Table 3:** Ablation experiments on the text feature extraction module based on the MPTD dataset.

Method	Param (M)	FLOPs (G)	Pancreas (%)	Tumor (%)	DSC (%)
LViT-NT	28.0	54.0	76.37	59.26	67.82
**Ours-NT**	**35.3**	**60.4**	**78.19**	**61.15**	**71.17**
LViT-WT	29.7	54.1	77.01	60.03	68.52
**Ours**	**65.6**	**63.2**	**79.25**	**64.21**	**71.73**

The best scores are highlighted.

#### 4.4.2 MTT and MEAM module

**Table 4** presents our proposed multimodal text-image fusion segmentation method with cross-modal reinforced attention (MEAM). The results demonstrate MEAM’s consistent performance gains for both BERT and AMBERT architectures, highlighting its generalization capability and robustness. Notably, the AMBERT+MEAM configuration achieves the maximum improvement of 2.61% in tumor Dice score over the BERT baseline. The integrated multimodal fusion approach (AMBERT+MEAM+MTT) delivers a 4.55% enhancement in tumor segmentation Dice score compared to the baseline.

**Table 4:** Ablation experiments on the MEAM module based on the MPTD dataset.

Method	FLOPs (G)	Pancreas (%)	Tumor (%)
BERT	54.1	77.01	60.03
BERT+MEAM	57.3	76.79	61.27
AMBERT	60.4	76.16	60.51
AMBERT+MEAM	68.1	78.04	62.27
AMBERT+MEAM+MTT	63.2	**79.25**	**64.21**

The best scores are highlighted.

**Figure 7:**
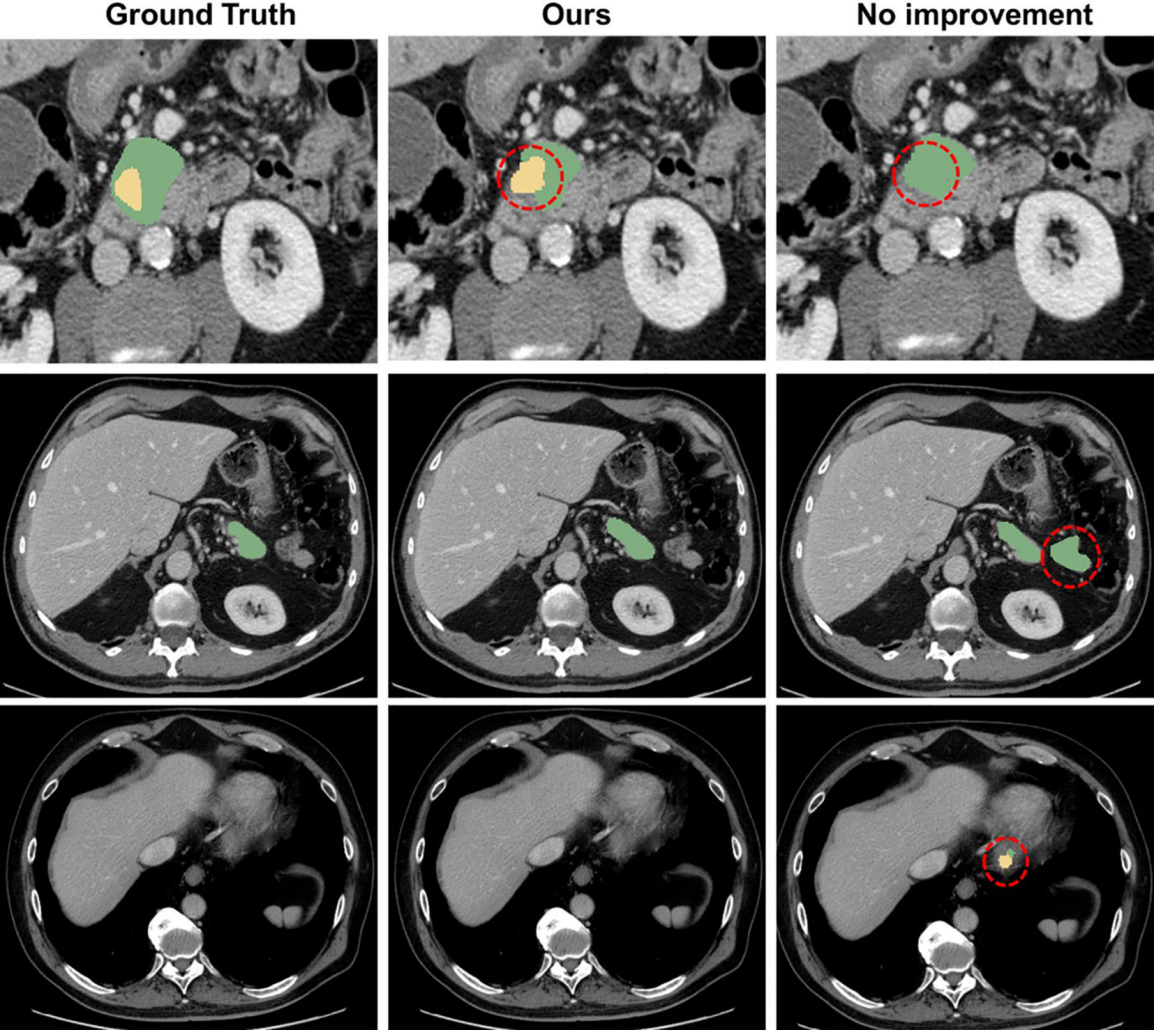
Comparison with the mis segmentation of original model. The pancreas is marked in green, and the tumor is marked in yellow. Red dashed circles indicate areas of incorrect segmentation.

Moreover, the integration of MEAM and MTT significantly enhances the feature extraction capability of the model, enabling it to focus more effectively on the morphology and boundary features of the pancreas and tumors. This improvement allows the model to more accurately capture tumor characteristics in complex medical image backgrounds and avoid incorrect predictions (as shown in **Figure 7**).

#### 4.4.3 Loss weight settings for DAS-Net

**Table 5** presents an ablation study on the loss weight configurations (λ_1_, λ_2_, λ_3_) in DAS-Net. The results demonstrate that the baseline configuration (0.5, 0.2, 0.05) achieves superior Dice scores for both pancreatic parenchyma and tumor lesions, validating the efficacy of this weighting scheme in enhancing segmentation performance.​

**Table 5:** Ablation experiments on the loss weight settings for DAS-Net based on the MPTD dataset.

( $\lambda_{1},\;\lambda_{2},\;\lambda_{3}$ )	Label ratio (%)	DSC (%)	Description
(0.5, 0.5, 0.05)	50	63.79	Noisy pseudo-label amplification
(0.2, 0.2, 0.05)	50	64.58	Underutilized unlabeled data
(0.8, 0.2, 0.05)	50	66.21	Fine-detail loss
(0.5, 0.2, 0.1)	50	67.15	Adversarial training saturation
(0.5, 0.2, 0.05)	50	**67.61**	**Baseline performance**

The best scores are highlighted.

### 4.5 Generalization experiment

To further validate our model’s generalizability and the effectiveness of incorporating textual information for improved segmentation accuracy, we conducted additional experiments on the QaTa-COV19 and MosMedData+ datasets. The QaTa-COVID-19 dataset (61), developed by researchers from Qatar University and Tampere University, comprises 9,258 COVID-19 chest X-ray images. The MosMedData dataset (62) contains 2,729 CT scan slices of lung infections.

To further validate the generalization of our model and assess how the introduction of text information enhances segmentation accuracy, we conducted additional experiments on the QaTa-COV19 and MosMedData datasets. These datasets are publicly available and were previously described. In these experiments, our model was compared against the current state-of-the-art (SOTA) methods, encompassing five fully supervised single-modal and five fully supervised multimodal segmentation methods.

**Figure 8:**
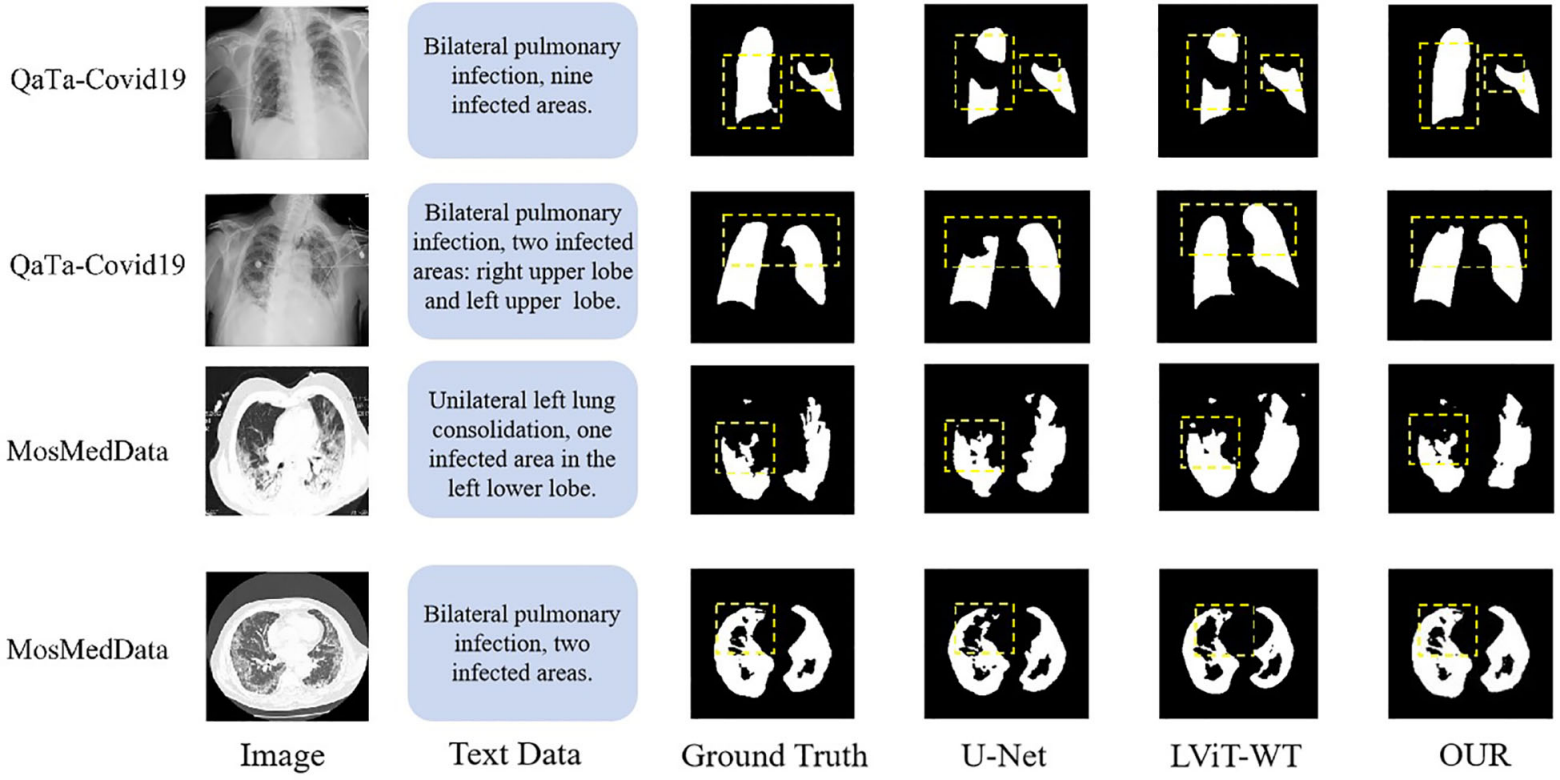
Visualization of QaTa-Covid19 and MosMedData dataset.

As shown in **Table 6**, on the QaTa-Covid19 dataset, our model improved the Dice score by 5.36% and the MIoU score by 5.64% over the second-best-performing nnUNet model. Remarkably, even with only 25% of training labels, our model still exceeded the performance of other state-of-the-art methods. These results underscore the critical role of textual information in enhancing model performance beyond unimodal approaches. Additionally, the MTT fusion module—which combines coarse and fine-grained text features—exhibited exceptional effectiveness, with our model achieving a 2.12% higher Dice score and a 4.66% higher MIoU score than LViT and LViT-TW, respectively. The corresponding visualization is provided in **Figure 8**.

**Table 6:** On the QaTa-Covid19 dataset, our model’s semi-supervised and fully supervised comparative experiments with state-of-the-art single-modal and multi-modal segmentation methods.

Method	Label ratio (%)	DSC (%)	MIoU (%)	95HD (Voxel)	MAE (Voxel)
U-Net	100	79.02	69.46	8.93	0.0875
UNet++	100	79.62	70.25	8.48	0.0663
nnUNet	100	80.42	70.81	7.02	0.0206
TransUNet	100	78.63	69.13	7.53	0.0219
Swin-Unet	100	78.07	68.34	7.07	0.0238
C2FVL	100	78.45	69.14	6.92	0.0543
CLIP	100	79.81	70.66	8.70	0.0637
ViLT	100	79.63	70.12	6.79	0.0712
LViT-NT	100	81.12	71.37	6.28	0.0182
LViT-WT	100	83.66	75.11	5.70	0.0139
**Ours**	**100**	**85.78**	**76.45**	**5.15**	**0.0096**
LViT-WT	25	80.88	71.98	5.75	0.0463
**Ours**	**25**	**81.79**	**73.06**	**5.40**	**0.0121**

The best scores are highlighted.

**Table 7** shows that the segmentation metrics on the MosMedData dataset are lower compared to those on the QaTa-Covid19 dataset, which may be due to the smaller size of the MosMedData dataset, which is about a quarter of the sample size of the QaTa-Covid19 data. This result shows the necessity of improving the text feature extraction and fusion methods. Comparative experiments with multimodal segmentation methods show that all fully supervised methods on the MosMedData dataset achieve scores of more than 70%, confirming the importance of text features to segmentation performance. However, our method is 4.16% higher than the LViT method and more than 6.73% higher than the CLIP text feature extraction method, which verifies the effectiveness of using coarse-grained and fine-grained text features to enhance the segmentation results. The visualization results are shown in **Figure 8**.

**Table 7:** On the MosMedData dataset, our model’s semi-supervised and fully supervised comparative experiments with state-of-the-art single-modal and multi-modal segmentation methods.

Method	Label ratio (%)	DSC (%)	MIoU (%)	95HD (Voxel)	MAE (Voxel)
U-Net	100	64.60	50.73	9.13	0.0275
UNet++	100	71.75	58.39	8.98	0.0263
nnUNet	100	72.59	60.36	7.55	0.0363
TransUNet	100	71.24	58.44	7.69	0.0212
Swin-Unet	100	63.29	50.19	7.72	0.0275
C2FVL	100	72.21	59.52	6.81	0.0301
CLIP	100	71.97	59.64	6.89	0.0916
ViLT	100	72.36	60.15	6.80	0.0212
LViT-NT	100	72.58	60.40	6.48	0.0263
LViT-WT	100	74.57	61.33	5.79	0.0916
**Ours**	**100**	**78.70**	**64.17**	**4.83**	**0.0168**
LViT-WT	25	70.58	61.40	5.92	0.0241
**Ours**	**25**	**73.70**	**63.17**	**5.54**	**0.0255**

The best scores are highlighted.

## 5 Discussion

### 5.1 Innovative aspects of SMF-Net

This work proposes SMF-Net, a novel dual-path CNN-Transformer architecture for accurate pancreas tumor segmentation by effectively integrating visual and textual modalities. The architecture combines a U-shaped CNN pathway with a Multimodal Transformer (MTT) branch to facilitate enhanced cross-modal feature fusion. A key innovation is the Multimodal Enhanced Attention Module (MEAM), embedded at CNN skip connections, which balances complementary image-text information while preserving critical anatomical details.

To address challenges of limited annotated data in medical imaging, we develop DAS-Net, a semi-supervised dual-learner adversarial framework that synergistically integrates consistency regularization with adversarial training to maximize unlabeled data utilization and improve model generalizability. Furthermore, we curate a clinically annotated multimodal dataset containing paired CT scans and diagnostic reports from 86 pancreatic cancer patients, providing valuable training resources. Extensive evaluations on this dataset as well as cross-dataset validations on QaTa-COV19 and MosMedData demonstrate SMF-Net’s state-of-the-art segmentation performance and robust generalization, highlighting its practical clinical potential.

### 5.2 Limitations and future work

#### 5.2.1 Training data dependence

Although our model demonstrates strong performance, its effectiveness on certain challenging cases—such as patients with organ deformities or tumor metastases—falls short compared to results on public datasets. This limitation likely stems from the inherent dependency of Transformer-based architectures on large-scale annotated data for optimal training and convergence. Given the scarcity of extensive labeled datasets in medical imaging, the model’s generalization capacity is constrained. Future research should focus on improving robustness and generalizability through approaches like self-supervised learning or advanced data augmentation methods that reduce reliance on annotated samples. Moreover, future work will aim to generalize SMF-Net to diverse imaging and text modalities, enhance its adaptability for multi-organ segmentation, and extend its application to broader tumor segmentation tasks to better support clinical decision-making.

#### 5.2.2 Feasibility of clinical application

The current SMF-Net framework requires textual input during inference, restricting its practical deployment scenarios. To overcome this limitation, future work could explore integrating large language models to automatically generate relevant textual annotations from image data. This advancement would enable fully automated multimodal inference without manual text input, thereby broadening the model’s applicability and usability in real-world clinical environments.

## 6 Conclusion

Given the diagnostic significance of CT imaging and pathology report text in clinical practice, we present a novel multimodal hybrid CNN-Transformer architecture, termed the Semantic-Guided Multimodal Fusion Network (SMF-NET), for simultaneous pancreas and tumor segmentation. To integrate textual and visual features, we introduce a Multimodal Text-Transformer (MTT) module that strengthens text feature extraction while highlighting semantic correlations between textual and imaging data. A dual-modality cross-attention module is further designed to maximize feature preservation by equally weighting contributions from both modalities. We also propose a Dual Adversarial Student Network (DAS-Net) framework for knowledge distillation and curate a multimodal pancreatic tumor dataset (MPTD) tailored for segmentation tasks. Extensive evaluations on an in-house MPTD dataset (86 patients) demonstrate SMF-NET’s superior pancreatic segmentation performance across varying training data partitions. Additional validation on the QaTa-COVID-19 and MosMedData lung datasets confirms its generalizability for multimodal organ segmentation. Experimental results indicate that SMF-NET achieves precise delineation of both pancreatic and pulmonary structures, underscoring its potential for clinical deployment.

## Data Availability

The raw data supporting the conclusions of this article will be made available by the authors, without undue reservation.
